# Outcomes by Comorbidities with Tremelimumab and Durvalumab in the Randomized, Controlled Phase 3 HIMALAYA Study in Unresectable Hepatocellular Carcinoma

**DOI:** 10.1159/lic/admag001

**Published:** 2026-07-28

**Authors:** Lorenza Rimassa, Farshid Dayyani, Robin K. Kelley, Bruno Sangro, Stephen L. Chan, Hitomi Nakamura, Lucy Clark, Ioannis Xynos, Ghassan K. Abou-Alfa

**Affiliations:** a Department of Biomedical Sciences, Humanitas University, Pieve Emanuele, Italy; b Hepatopancreatobiliary Oncology Unit, Humanitas Cancer Center, IRCCS Humanitas Research Hospital, Rozzano, Italy; c Department of Medicine, Division of Hematology/Oncology, University of California Irvine, Orange, CA, USA; d Helen Diller Family Comprehensive Cancer Center, University of California, San Francisco, CA, USA; e Liver Unit and HPB Oncology Area, Clínica Universidad de Navarra and CIBEREHD, Pamplona, Spain; f State Key Laboratory of Translational Oncology, Department of Clinical Oncology, Sir Yue-Kong Pao Center for Cancer, Prince of Wales Hospital, The Chinese University of Hong Kong, Hong Kong, Hong Kong SAR; g Global Medical Affairs, AstraZeneca, Cambridge, UK; h Oncology R&D, AstraZeneca, Cambridge, UK; i Oncology R&D, Late-Stage Development, AstraZeneca, Cambridge, UK; j Department of Medicine, Memorial Sloan Kettering Cancer Center, Cornell University, New York, NY, USA; k Weill Medical College, Cornell University, New York, NY, USA; l Trinity College Dublin, Dublin, Ireland

**Keywords:** Comorbidities, Durvalumab, Immune checkpoint inhibitor, Tremelimumab, Unresectable hepatocellular carcinoma

## Abstract

**Introduction:**

People with hepatocellular carcinoma (HCC) often have underlying comorbidities, which may impact prognosis and lead to safety challenges following treatment. This exploratory, post hoc analysis of the phase 3 HIMALAYA study (NCT03298451) assessed outcomes in participants with unresectable HCC and cardiovascular or metabolic comorbidities at baseline.

**Methods:**

Participants with unresectable HCC were randomized to STRIDE (Single Tremelimumab Regular Interval Durvalumab), durvalumab monotherapy, or sorafenib. Comorbidities were based on medical history assessed at baseline and qualified using preferred terms from cardiovascular or metabolic Standardised Medical Dictionary for Regulatory Activities Queries. Overall survival (OS) rates were evaluated in participants with or without cardiovascular or metabolic comorbidities within and by treatment group. The proportion of long-term survivors (alive ≥36 months post-randomization) and safety were assessed in participants with cardiovascular or metabolic comorbidities. Data cutoffs: August 27, 2021 (primary analysis); January 23, 2023 (4-year updated analysis, OS, and serious treatment-related adverse events).

**Results:**

Hypertension and type 2 diabetes mellitus were the most common cardiovascular and metabolic comorbidities, respectively. The 48-month OS rates with STRIDE were 28.0% and 21.7% in participants with and without cardiovascular comorbidities and 20.4% and 28.5% in those with and without metabolic comorbidities, respectively. In total, 28.2% of participants with cardiovascular comorbidities and 22.6% of participants with metabolic comorbidities experienced long-term survival with STRIDE. Safety in participants with cardiovascular or metabolic comorbidities was generally consistent with all participants in the safety analysis set; no new safety signals were observed.

**Conclusions:**

OS benefits with STRIDE were similar in participants with vs. without cardiovascular or metabolic comorbidities, with manageable safety in participants with comorbidities. These results support the durable benefits of STRIDE in a broad population reflective of unresectable HCC globally.

## Introduction

Hepatocellular carcinoma (HCC) is the most common form of primary liver cancer and is one of the leading contributors to cancer-related deaths globally [1–3]. Most people with HCC are typically diagnosed at an older age, when comorbidities are common, and at an advanced stage of the disease [4–6]. The global incidence of HCC is increasing and the underlying etiology leading to HCC varies geographically [1, 7].

Approximately 90% of HCC cases are associated with underlying chronic liver disease [1]. Hepatitis B virus (HBV) accounts for approximately 60% of HCC cases and is the predominant etiology of HCC in most parts of Asia, excluding Japan, and Africa, whereas hepatitis C virus (HCV) or nonviral etiologies are more common in Japan and Western countries [1, 8]. The incidence of nonviral etiologies of HCC, such as metabolic dysfunction-associated steatotic liver disease (MASLD), is on the rise and is expected to increase worldwide [9, 10]. Risk factors for MASLD, such as old age, diabetes, and hypertension, which are associated with an increased risk of cardiovascular disease, are also risk factors for HCC [11]. These risk factors are closely related, in that they are also associated with metabolic dysfunction, which may impact prognosis and lead to safety concerns during treatment [12, 13].

The management of HCC is complex since people may be impacted by the burden of the underlying chronic liver disease in addition to the cancer [14]. Comorbidities may affect the various stages of the cancer patient journey from diagnosis, through treatment, to outcomes [15]. Although treatment decision-making in HCC has always required assessment of the extent of the underlying liver disease, historical treatment algorithms adopt a cancer-focused treatment approach, not consistently addressing the interrelations between cancer and all comorbidities [15–17]. More recently, contemporary clinical practice guidelines have come to regard comorbidities as one of the key determinants of clinical decision-making for the management of HCC [7].

At the primary analysis of the phase 3 HIMALAYA study (NCT03298451) in unresectable HCC, the STRIDE (Single Tremelimumab Regular Interval Durvalumab) regimen significantly improved overall survival (OS) vs. sorafenib as demonstrated by an OS hazard ratio (HR) from a stratified Cox proportional hazards model of 0.78 (96.02% confidence interval [CI]: 0.65–0.93; stratified log-rank test *p* = 0.0035) [18]. Durvalumab monotherapy was non-inferior to sorafenib for OS [18]. The 4- and 5-year updated analyses of the HIMALAYA study confirmed a durable OS benefit with STRIDE vs. sorafenib (HR, 0.78; 95% CI, 0.67–0.92; and HR, 0.76; 95% CI, 0.65–0.89, respectively) [19, 20]. The 4- and 5-year OS HR (95% CI) for durvalumab monotherapy vs. sorafenib were 0.86 (0.74–1.01) and 0.85 (0.73–1.00), respectively [19, 20]. The 48- and 60-month OS rates at the 4- and 5-year updated analyses were 25.2% and 19.6% with STRIDE, 19.3% and 14.4% with durvalumab monotherapy, and 15.1% and 9.4% with sorafenib, respectively [19, 20]. At the 5-year updated analysis, there were no new serious treatment-related adverse events (TRAEs) with STRIDE, and the safety profile was both manageable and consistent with the primary analysis [18, 20]. Based on these results, STRIDE has been approved globally for the treatment of adults with advanced and/or unresectable HCC, including across North America, Europe, and parts of the Asia-Pacific region [21–25]. Durvalumab monotherapy has been approved for the treatment of adults with unresectable HCC in Europe and Japan [24, 26].

With the changing epidemiologic landscape for HCC, studies evaluating the effect of cardiovascular and metabolic comorbidities on the outcomes of immunotherapy for people with unresectable HCC are needed. This post hoc analysis aimed to assess the presence and potential impact of cardiovascular and metabolic comorbidities on the efficacy and safety of STRIDE and durvalumab monotherapy in participants with unresectable HCC in the HIMALAYA study.

## Methods

### Study Design and Participants

HIMALAYA (ClinicalTrials.gov, NCT03298451) was a randomized, open-label, multicenter, global, phase 3 study [18]. The HIMALAYA study design and methodology have been reported in detail previously [18]. Eligible study participants included adults aged ≥18 years with histologically confirmed unresectable HCC, no prior systemic therapy and who were not eligible for locoregional therapy. Participants also had Barcelona Clinic Liver Cancer stage B, unsuitable for locoregional therapies, or stage C, Child-Pugh Class A, Eastern Cooperative Oncology Group (ECOG) performance status of 0 or 1, and at least one measurable lesion per Response Evaluation Criteria in Solid Tumors, version 1.1. Exclusion criteria included clinically meaningful ascites (defined as ascites requiring nonpharmacologic intervention), main portal vein thrombosis or inferior vena cava thrombosis, and coinfection with HBV and HCV. Therapeutic anticoagulation as prophylaxis for indications such as atrial fibrillation in participants without liver cirrhosis was permitted. Endoscopy was not required at enrollment unless otherwise stipulated by the investigator in participants at high risk of gastrointestinal bleeding.

Participants were randomly assigned 1:1:1 to receive STRIDE (tremelimumab 300 mg [one dose] plus durvalumab 1,500 mg every 4 weeks), durvalumab monotherapy (1,500 mg every 4 weeks), or sorafenib (400 mg twice daily). Randomization was stratified according to macrovascular invasion (MVI; yes or no), etiology of liver disease (HBV or HCV [but not both] or other/nonviral), and ECOG performance status 0 or 1.

In the present exploratory, post hoc analysis, participants were evaluated for efficacy and safety outcomes by the presence of cardiovascular or metabolic comorbidities at baseline. Cardiovascular (including hypertension) and metabolic (including diabetes mellitus/hyperglycemia) comorbidities were based on medical history reported at baseline and qualified using preferred terms from cardiovascular or metabolic Standardised Medical Dictionary for Regulatory Activities Queries (all preferred terms used are listed in the online suppl. Table S1, S2; for all online suppl. material, see https://doi.org/10.1159/lic/admag001). Participants with >1 type of comorbidity were counted once in each category. Participants with both cardiovascular and metabolic comorbidities were included in both subgroups, respectively. The HIMALAYA study was conducted in accordance with the Declaration of Helsinki, International Council for Harmonisation Guideline for Good Clinical Practice, applicable regulatory requirements, and the AstraZeneca Bioethics Policy. The trial protocol was approved by local Institutional Review Boards. Written, informed consent was obtained from participants or their legal representatives before participation.

### Outcomes

This exploratory, post hoc analysis was based on the primary data cutoff (August 27, 2021), with the exception of the analysis of OS and serious TRAEs, which was based on the 4-year data cutoff (January 23, 2023) [19]. OS by comorbidities was assessed in the full analysis set (participants randomized to STRIDE, durvalumab monotherapy, or sorafenib), including assessment of long-term survivors (participants alive ≥36 months beyond randomization). Landmark OS rates at 36 and 48 months and within-arm OS HRs and 95% CIs were calculated. Safety was assessed by the occurrence of adverse events (AEs) in the safety analysis set (participants who received ≥1 dose of study drug).

### Statistical Analysis

In this post hoc analysis, baseline characteristics were summarized descriptively by treatment arm for each comorbidity subgroup in the full analysis set. OS HRs with corresponding 95% CIs were estimated using a Cox proportional hazards model with the Efron method for handling ties. Median OS and landmark survival rates were calculated using the Kaplan-Meier technique. To account for potential confounding by baseline factors, multivariable Cox regression was performed to estimate adjusted OS HRs between comorbidity groups within each treatment arm. Models were adjusted for baseline prognostic factors that were imbalanced between comorbidity subgroups (ECOG performance status, MVI, extrahepatic spread, alpha-fetoprotein, and albumin-bilirubin score) separately for the cardiovascular and metabolic subgroups. Variable selection was guided by clinical relevance and baseline imbalance, and stepwise regression was applied, with the final model and covariates chosen using the Bayesian Information Criterion. The interaction between comorbidity subgroup and treatment on OS was assessed using a Cox proportional hazard regression model that included treatment, baseline value of the comorbidity subgroup, and their interaction term. *P* values (2-sided) for the interaction term were used to assess whether treatment effect on survival varied by the presence of comorbidities. To control the family-wise error rates across interaction tests, multiplicity *p* values were also calculated using the Holm step-down procedure. Percentages were calculated from the number of participants with comorbidities of interest in the full analysis set. Safety was summarized descriptively for each subgroup by actual treatment received using the safety analysis set.

## Results

### Participant Demographics and Clinical Characteristics

Of 1,171 global participants with unresectable HCC, a total of 1,019 (87.0%) participants across the STRIDE, durvalumab monotherapy, and sorafenib arms had cardiovascular or metabolic comorbidities. Cardiovascular comorbidities were reported in 216 (55.0%) participants in the STRIDE arm, 172 (44.2%) participants in the durvalumab monotherapy arm, and 207 (53.2%) participants in the sorafenib arm (Table 1). Metabolic comorbidities were reported in 159 (40.5%) participants in the STRIDE arm, 130 (33.4%) participants in the durvalumab monotherapy arm, and 135 (34.7%) participants in the sorafenib arm (Table 1). Overall, 319 (27.2%) participants had both cardiovascular and metabolic comorbidities: 123 (31.3%) in the STRIDE arm, 90 (23.1%) in the durvalumab monotherapy arm, and 106 (27.2%) in the sorafenib arm.

**Table 1.** Baseline demographics and disease characteristics in participants with and without cardiovascular or metabolic comorbidities

​	STRIDE (*n =* 393)				Durvalumab (*n =* 389)				Sorafenib (*n =* 389)			
	cardiovascular comorbidities		metabolic comorbidities		cardiovascular comorbidities		metabolic comorbidities		cardiovascular comorbidities		metabolic comorbidities	
	yes (*n =* 216)	no (*n =* 177)	yes (*n =* 159)	no (*n =* 234)	yes (*n =* 172)	no (*n =* 217)	yes (*n =* 130)	no (*n =* 259)	yes (*n =* 207)	no (*n =* 182)	yes (*n =* 135)	no (*n =* 254)
Median age (range), years	69 (36–84)	59 (22–86)	69 (39–84)	62 (22–86)	68 (40–86)	60 (20–86)	67 (47–86)	61 (20–86)	68 (45–88)	60 (18–82)	68 (45–86)	63 (18–88)
Male sex, *n* (%)	176 (81.5)	151 (85.3)	133 (83.6)	194 (82.9)	143 (83.1)	180 (82.9)	114 (87.7)	209 (80.7)	185 (89.4)	152 (83.5)	125 (92.6)	212 (83.5)
Asia region, *n* (%)^a^	68 (31.5)	88 (49.7)	58 (36.5)	98 (41.9)	49 (28.5)	118 (54.4)	39 (30.0)	128 (49.4)	58 (28.0)	98 (53.8)	40 (29.6)	116 (45.7)
ECOG PS, *n* (%)												
0	123 (56.9)	121 (68.4)	96 (60.4)	148 (63.2)	96 (55.8)	141 (65.0)	78 (60.0)	159 (61.4)	119 (57.5)	122 (67.0)	87 (64.4)	154 (60.6)
1	92 (42.6)	56 (31.6)	62 (39.0)	86 (36.8)	75 (43.6)	75 (34.6)	52 (40.0)	98 (37.8)	88 (42.5)	59 (32.4)	48 (35.6)	99 (39.0)
2	1 (0.5)	0	1 (0.6)	0	1 (0.6)	1 (0.5)	0	2 (0.8)	0	1 (0.5)	0	1 (0.4)
BCLC score, *n* (%)												
B	44 (20.4)	33 (18.6)	40 (25.2)	37 (15.8)	39 (22.7)	41 (18.9)	32 (24.6)	48 (18.5)	43 (20.8)	23 (12.6)	24 (17.8)	42 (16.5)
C	172 (79.6)	144 (81.4)	119 (74.8)	197 (84.2)	133 (77.3)	176 (81.1)	98 (75.4)	211 (81.5)	164 (79.2)	159 (87.4)	111 (82.2)	212 (83.5)
Viral etiology, *n* (%)												
HBV^b^	49 (22.7)	73 (41.2)	39 (24.5)	83 (35.5)	32 (18.6)	87 (40.1)	25 (19.2)	94 (36.3)	42 (20.3)	77 (42.3)	24 (17.8)	95 (37.4)
HCV^c^	57 (26.4)	53 (29.9)	28 (17.6)	82 (35.0)	48 (27.9)	59 (27.2)	29 (22.3)	78 (30.1)	55 (26.6)	49 (26.9)	22 (16.3)	82 (32.3)
Nonviral^d^	110 (50.9)	51 (28.8)	92 (57.9)	69 (29.5)	92 (53.5)	71 (32.7)	76 (58.5)	87 (33.6)	110 (53.1)	56 (30.8)	89 (65.9)	77 (30.3)
MVI, *n* (%)	58 (26.9)	45 (25.4)	44 (27.7)	59 (25.2)	42 (24.4)	52 (24.0)	33 (25.4)	61 (23.6)	51 (24.6)	49 (26.9)	44 (32.6)	56 (22.0)
EHS, *n* (%)	112 (51.9)	97 (54.8)	73 (45.9)	136 (58.1)	85 (49.4)	127 (58.5)	56 (43.1)	156 (60.2)	97 (46.9)	106 (58.2)	64 (47.4)	139 (54.7)
ALBI score, *n* (%)												
1	115 (53.2)	102 (57.6)	80 (50.3)	137 (58.5)	84 (48.8)	114 (52.5)	50 (38.5)	148 (57.1)	102 (49.3)	101 (55.5)	69 (51.1)	134 (52.8)
2	99 (45.8)	75 (42.4)	79 (49.7)	95 (40.6)	87 (50.6)	102 (47.0)	79 (60.8)	110 (42.5)	105 (50.7)	80 (44.0)	66 (48.9)	119 (46.9)
3	1 (0.5)	0	0	1 (0.4)	1 (0.6)	1 (0.5)	1 (0.8)	1 (0.4)	0	1 (0.5)	0	1 (0.4)
Missing	1 (0.5)	0	0	1 (0.4)	0	0	0	0	0	0	0	0

Data cutoff: August 27, 2021.

ALBI, albumin-bilirubin; BCLC, Barcelona Clinic Liver Cancer; ECOG, Eastern Cooperative Oncology Group; EHS, extrahepatic spread; HBV, hepatitis B virus; HCV, hepatitis C virus; MVI, macrovascular invasion; PS, performance status; STRIDE, Single Tremelimumab Regular Interval Durvalumab.

^a^ Excluding Japan.

^b^ Tested positive for hepatitis B surface antigen and/or anti-hepatitis B core antibody with detectable HBV DNA.

^c^ Tested positive for HCV or had a history of HCV infection.

^d^ No active viral hepatitis identified.

Across treatment arms, hypertension was the most common cardiovascular comorbidity (93.4%), followed by peripheral edema (2.7%) and myocardial fibrosis (2.5%). Type 2 diabetes mellitus was the most common metabolic comorbidity across treatment arms (77.1%), followed by dyslipidemia (17.9%), hyperlipidemia (12.0%), and hypercholesterolemia (9.4%) (online suppl. Tables S1, S2).

Participant baseline demographics and disease characteristics were generally balanced across treatment arms in each cardiovascular or metabolic comorbidity subgroup (Table 1). Participants with cardiovascular or metabolic comorbidities were older than those without the respective comorbidities in the STRIDE, durvalumab monotherapy, and sorafenib arms. Greater proportions of participants with cardiovascular or metabolic comorbidities in the STRIDE, durvalumab monotherapy, and sorafenib arms were not from the Asian region and had nonviral underlying etiology vs. those without the respective comorbidities, who were predominantly from the Asia region (excluding Japan) with underlying HBV or HCV etiology. Between 55.8% and 68.4% of participants with and without cardiovascular or metabolic comorbidities had an ECOG performance status of 0 at baseline.

### Efficacy

Landmark OS rates at 36 and 48 months were generally similar (<10.0% difference) between participants with and without cardiovascular or metabolic comorbidities across the STRIDE, durvalumab monotherapy, and sorafenib arms (Table 2) and were consistent with trends in landmark OS rates observed in the full analysis set [19]. In participants with vs. without cardiovascular comorbidities, within-arm OS HRs (95% CI) were 0.86 (0.68–1.08) for STRIDE (Fig. 1a), 0.98 (0.78–1.23) for durvalumab monotherapy (Fig. 1b), and 0.93 (0.74–1.16) for sorafenib (Fig. 1c). In participants with vs. without metabolic comorbidities, within-arm OS HRs (95% CI) were 1.16 (0.92–1.47) for STRIDE (Fig. 1d), 1.05 (0.83–1.33) for durvalumab monotherapy (Fig. 1e), and 0.86 (0.68–1.08) for sorafenib (Fig. 1f). The adjusted HRs from the multivariable analysis, comparing patients with vs. without cardiovascular and metabolic comorbidities within treatment arms, remained similar in direction and magnitude to the unadjusted estimates, suggesting there is still some residual confounding (online suppl. Table S3). The unadjusted and multiplicity-adjusted *p* values for the interaction between treatment and comorbidity subgroup were above the prespecified exploratory threshold of 0.05, indicating that the treatment effect did not vary by presence of comorbidities for OS (online suppl. Table S3). In total, 28.2%, 19.2%, and 16.4% of participants with cardiovascular comorbidities and 22.6%, 16.2%, and 15.6% of participants with metabolic comorbidities experienced long-term survival with STRIDE, durvalumab monotherapy, and sorafenib, respectively (Fig. 2).

**Table 2.** Landmark overall survival rates in participants with and without cardiovascular or metabolic comorbidities

​	STRIDE (*n =* 393)				Durvalumab (*n =* 389)				Sorafenib (*n =* 389)			
	cardiovascular comorbidities		metabolic comorbidities		cardiovascular comorbidities		metabolic comorbidities		cardiovascular comorbidities		metabolic comorbidities	
	yes (*n =* 216)	no (*n =* 177)	yes (*n =* 159)	no (*n =* 234)	yes (*n =* 172)	no (*n =* 217)	yes (*n =* 130)	no (*n =* 259)	yes (*n =* 207)	no (*n =* 182)	yes (*n =* 135)	no (*n =* 254)
OS rate, % (95% CI)												
36 months	32.9 (26.6–39.3)	28.0 (21.6–34.8)	25.7 (19.1–32.8)	34.0 (27.9–40.1)	24.2 (18.0–31.0)	24.9 (19.3–31.0)	21.9 (14.9–29.7)	26.0 (20.7–31.5)	18.7 (13.5–24.4)	21.4 (15.5–27.8)	21.1 (14.3–28.7)	19.1 (14.4–24.3)
Participants at risk, *n*	61	42	36	67	33	46	21	58	34	30	21	43
48 months	28.0 (22.0–34.2)	21.7 (15.6–28.5)	20.4 (14.3–27.2)	28.5 (22.7–34.6)	18.7 (12.9–25.3)	19.7 (14.4–25.5)	16.2 (9.9–23.8)	20.7 (15.8–26.1)	15.1 (10.4–20.6)	14.8 (9.3–21.5)	15.9 (9.9–23.2)	14.7 (10.3–19.8)
Participants at risk, *n*	35	19	21	33	17	22	12	27	19	9	9	19

Data cutoff: January 23, 2023.

CI, confidence interval; OS, overall survival; STRIDE, Single Tremelimumab Regular Interval Durvalumab.

**Fig. 1.**
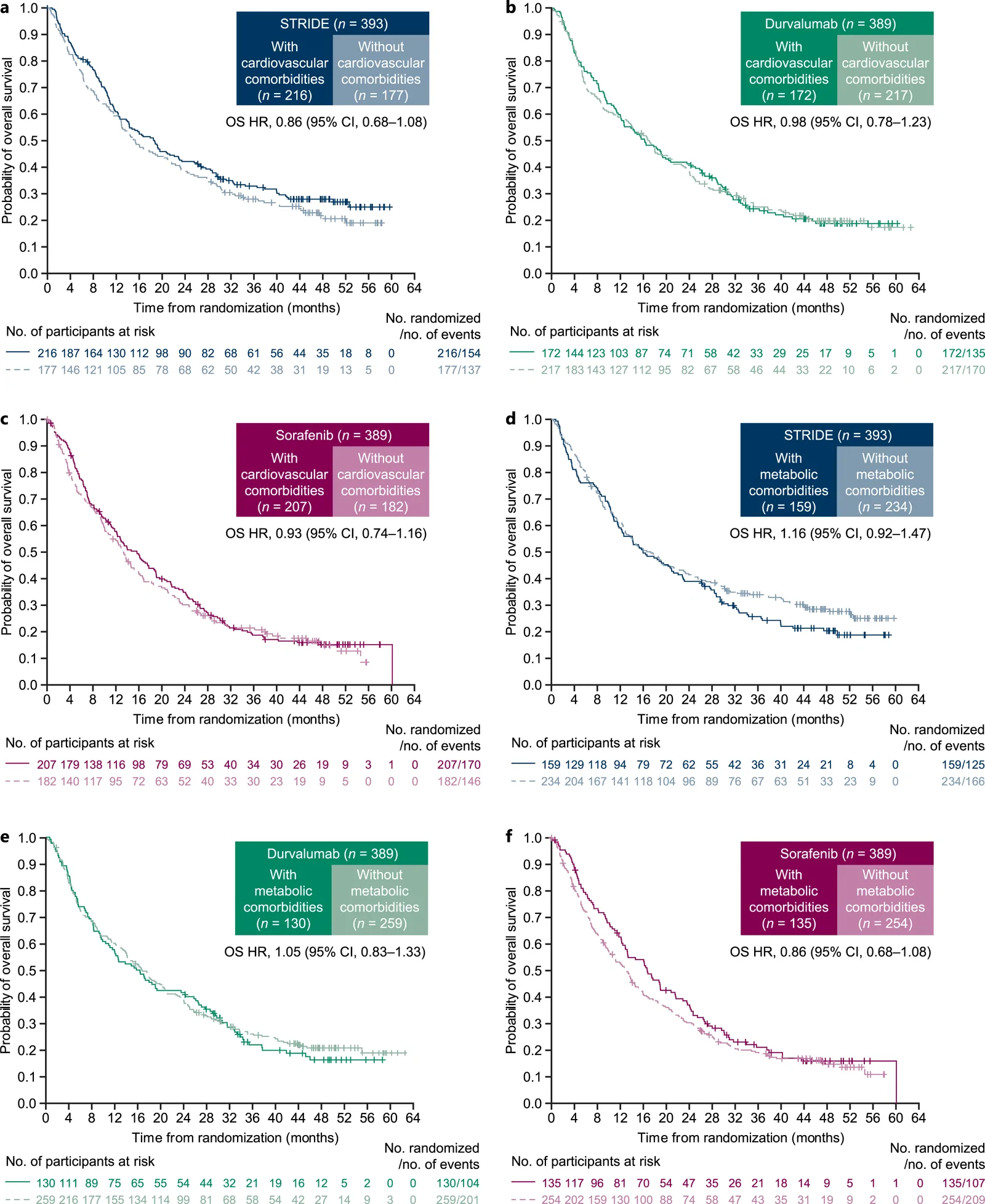
Overall survival (within-arm) for STRIDE (**a**), durvalumab (**b**), and sorafenib (**c**) in participants with and without cardiovascular comorbidities and STRIDE (**d**), durvalumab (**e**), and sorafenib (**f**) in participants with and without metabolic comorbidities. Data cutoff: January 23, 2023. CI, confidence interval; HR, hazard ratio; OS, overall survival; STRIDE, Single Tremelimumab Regular Interval Durvalumab.

**Fig. 2.**
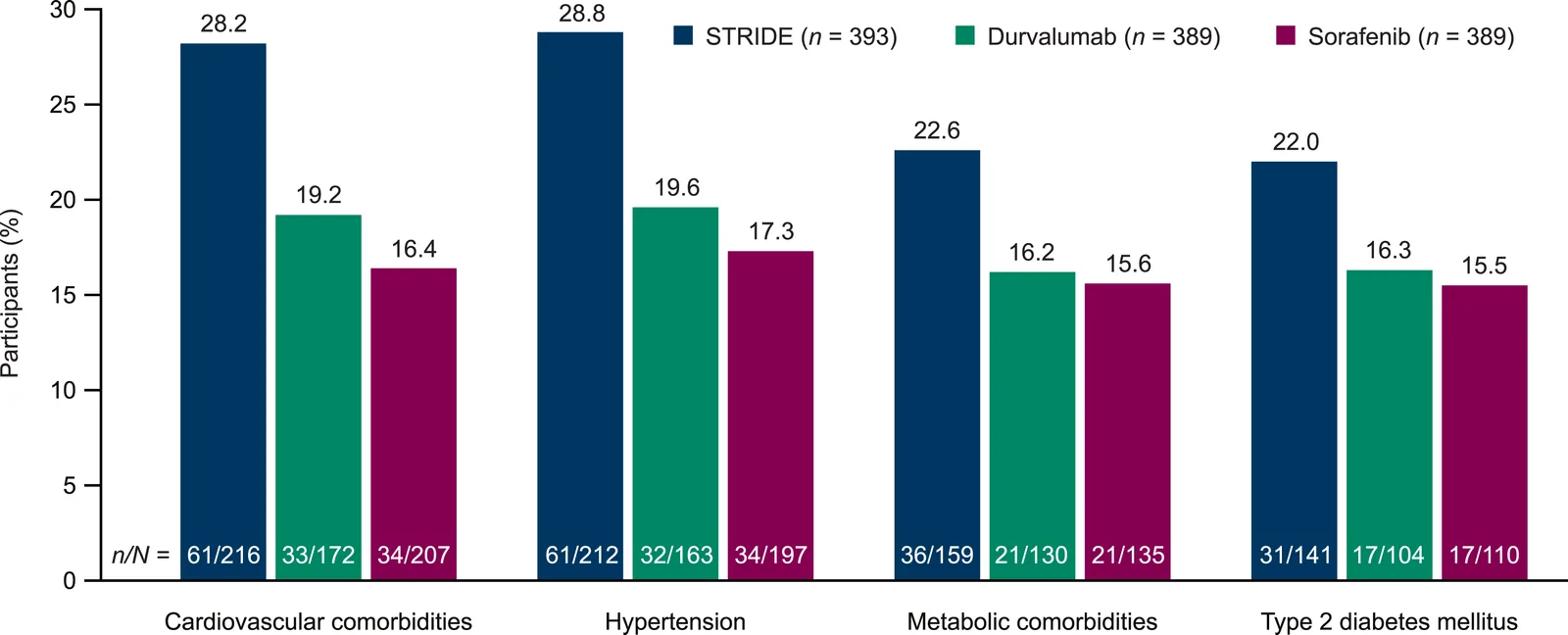
Proportion of long-term survivors^a^ in participants with comorbidities. Data cutoff: January 23, 2023. ^a^Participants alive ≥36 months beyond randomization. Percentages were calculated from the number of participants with comorbidities of interest in the full analysis set. *n,* number of long-term survivors; *N*, total number of participants with comorbidity of interest in the full analysis set; STRIDE, Single Tremelimumab Regular Interval Durvalumab.

### Safety

At the primary data cutoff, AEs were reported in 99.1%, 89.5%, and 96.0% of participants with cardiovascular comorbidities, and in 98.7%, 89.4%, and 96.2% of participants with metabolic comorbidities, in the STRIDE, durvalumab monotherapy, and sorafenib arms, respectively (Table 3). In participants with cardiovascular comorbidities, TRAEs were reported in 80.9% of participants in the STRIDE arm, 56.4% of participants in the durvalumab monotherapy arm, and 86.6% of participants in the sorafenib arm (Table 3). In participants with metabolic comorbidities, 80.3% in the STRIDE arm, 53.0% in the durvalumab monotherapy arm, and 87.8% in the sorafenib arm reported TRAEs (Table 3). Grade 3 or 4 TRAEs were reported in 29.3%, 13.4%, and 39.3% of participants with cardiovascular comorbidities and in 29.3%, 18.2%, and 41.2% of participants with metabolic comorbidities, receiving STRIDE, durvalumab monotherapy, and sorafenib, respectively (Table 3). TRAEs leading to death occurred in 2.8% and 1.0% of participants with cardiovascular comorbidities receiving STRIDE and sorafenib, respectively, and in 3.8% and 1.5% of participants with metabolic comorbidities receiving STRIDE and sorafenib, respectively; no TRAEs leading to death occurred in the durvalumab monotherapy arm in participants with cardiovascular or metabolic comorbidities (Table 3). TRAEs leading to discontinuation of study treatment were reported in 10.2%, 4.1%, and 12.9% of participants with cardiovascular comorbidities and in 10.2%, 5.3%, and 11.5% of participants with metabolic comorbidities, receiving STRIDE, durvalumab monotherapy, and sorafenib, respectively (Table 3). The rates of grade 3 or 4 hemorrhagic or bleeding events in participants with cardiovascular or metabolic comorbidities were 4.2% and 3.2% with STRIDE, 2.3% and 1.5% with durvalumab monotherapy, and 4.5% and 6.1% with sorafenib (online suppl. Table S4). The rates of grade 3 or 4 cardiovascular/hypertension events in participants with cardiovascular or metabolic comorbidities were 5.6% and 3.2% with STRIDE, 2.3% and 2.3% with durvalumab monotherapy, and 9.0% and 1.5% with sorafenib (online suppl. Table S5). The rates of grade 3 or 4 metabolic events in participants with cardiovascular or metabolic comorbidities were 2.3% and 4.5% with STRIDE, 2.3% and 4.5% with durvalumab monotherapy, and 2.0% and 3.1% with sorafenib (online suppl. Table S6). The incidence of immune-mediated AEs is provided in Table 3.

**Table 3.** Safety in participants with cardiovascular or metabolic comorbidities in the safety analysis set

​	STRIDE (*n =* 388)			Durvalumab (*n =* 388)			Sorafenib (*n =* 374)		
	cardiovascular comorbidities (*n =* 215)	metabolic comorbidities (*n =* 157)	safety analysis set (*n* = 388)^1^	cardiovascular comorbidities (*n =* 172)	metabolic comorbidities (*n =* 132)	safety analysis set (*n* = 388)^1^	cardiovascular comorbidities (*n =* 201)	metabolic comorbidities (*n =* 131)	safety analysis set (*n* = 374)^1^
Participants with event, *n* (%)									
Any AE	213 (99.1)	155 (98.7)	378 (97.4)	154 (89.5)	118 (89.4)	345 (88.9)	193 (96.0)	126 (96.2)	357 (95.5)
Any TRAE	174 (80.9)	126 (80.3)	294 (75.8)	97 (56.4)	70 (53.0)	202 (52.1)	174 (86.6)	115 (87.8)	317 (84.8)
Any grade 3 or 4 AE	121 (56.3)	91 (58.0)	196 (50.5)	70 (40.7)	63 (47.7)	144 (37.1)	112 (55.7)	76 (58.0)	196 (52.4)
Any grade 3 or 4 TRAE	63 (29.3)	46 (29.3)	100 (25.8)	23 (13.4)	24 (18.2)	50 (12.9)	79 (39.3)	54 (41.2)	138 (36.9)
Any serious TRAE	45 (20.9)^a^	33 (21.0)^a^	68 (17.5)	12 (7.0)^a^	16 (12.1)^a^	32 (8.2)	19 (9.5)^a^	14 (10.7)^a^	35 (9.4)
Any TRAE leading to death	6 (2.8)	6 (3.8)	9 (2.3)	0	0	0	2 (1.0)	2 (1.5)	3 (0.8)
Any TRAE leading to treatment discontinuation	22 (10.2)	16 (10.2)	32 (8.2)	7 (4.1)	7 (5.3)	16 (4.1)	26 (12.9)	15 (11.5)	41 (11.0)
Any imAE	86 (40.0)	60 (38.2)	139 (35.8)	32 (18.6)	29 (22.0)	64 (16.5)	16 (8.0)	8 (6.1)	30 (8.0)

Data cutoff: August 27, 2021 (unless indicated otherwise).

AE, adverse event; imAE, immune-mediated adverse event; ITT, intent-to-treat; STRIDE, Single Tremelimumab Regular Interval Durvalumab; TRAE, treatment-related adverse event.

^1^ Abou-Alfa et al. [18].

^a^ Data cutoff: January 23, 2023.

At the 4-year follow-up analysis, serious TRAEs occurred in 20.9%, 7.0%, and 9.5% of participants with cardiovascular comorbidities and in 21.0%, 12.1%, and 10.7% of participants with metabolic comorbidities, receiving STRIDE, durvalumab monotherapy, and sorafenib, respectively (Table 3).

## Discussion

HIMALAYA was a large global study that demonstrated a long-term OS benefit for participants treated with STRIDE compared with sorafenib across clinically relevant subgroups [18–20]. At the 4- and 5-year follow-up analyses, no new serious TRAEs occurred with STRIDE (17.5% [68/388] and 17.5% [68/388]) compared with durvalumab monotherapy (8.5% [33/388] and 8.5% [33/388]) and sorafenib (9.6% [36/374] and 9.9% [37/374]) since the primary analysis (17.5% [68/388], 8.2% [32/388], and 9.4% [35/374], respectively) [18–20]. Durvalumab monotherapy maintained OS non-inferiority to sorafenib across subgroups and no late-onset safety signals were identified at the 4- and 5-year updated analyses [19, 20].

Between 1991 and 2019, the prevalence of MASLD increased worldwide from 21.9% to 37.3% [27]. With obesity and type 2 diabetes mellitus on the rise, it is projected that up to 38% of all cases of HCC may be attributed to MASLD globally [28, 29]. Type 2 diabetes mellitus is an independent risk factor for HCC irrespective of MASLD [28]. While there is an association between MASLD and the components of metabolic syndrome, including hypertension, diabetes, obesity, and dyslipidemia, the relationship between cardiovascular disease and MASLD is poorly understood [30, 31]. However, studies have reported a higher risk of cardiovascular disease among people with MASLD who also have type 2 diabetes mellitus [12].

This exploratory, post hoc analysis of efficacy and safety in participants with cardiovascular or metabolic comorbidities in HIMALAYA continues to support the use of the STRIDE regimen in a broad patient population with unresectable HCC with comorbidities. Participants with cardiovascular or metabolic comorbidities, most of whom were not from the Asia region (excluding Japan), were more likely to have nonviral HCC compared with participants without comorbidities. This was an expected finding, which coincides both with the global trends in the shift in HCC etiology and with the nonviral etiology itself, as well as the westernization of diets and the growing global obesity and type 2 diabetes mellitus epidemics [1, 29]. In addition, participants with cardiovascular disorders were older than those without the respective comorbidity, which is consistent with the association between cardiovascular disease and advanced age demonstrated in previous studies [32, 33].

Notably, landmark OS rates for participants with cardiovascular comorbidities in the STRIDE group were numerically higher than for those without cardiovascular comorbidities, and the adjusted HR (95% CI) for STRIDE in participants with vs. without cardiovascular comorbidities was 0.73 (0.58–0.93). These findings are counterintuitive and may have resulted from selection bias or unmeasured confounding.

Historically, phase 3 studies of systemic therapies for the treatment of HCC exclude special populations comprising people with relevant and frequently occurring comorbidities [34]. For example, in HIMALAYA, participants were excluded if they had symptomatic or uncontrolled hypertension, autoimmune or inflammatory disorders, and uncontrolled inter-current illness such as symptomatic congestive heart failure, unstable angina pectoris, and cardiac arrhythmia. This indicates that the cardiovascular comorbidity subgroup might have been “healthier” than expected in real-world clinical settings. Additionally, compared with participants with cardiovascular comorbidities, those without were more likely to be from Asia, have HBV-related HCC and possibly possess more aggressive tumors, as suggested by the higher proportion with extrahepatic spread. Furthermore, participants with cardiovascular comorbidities may benefit from enhanced health surveillance, or their tumor biology could differ, which might explain the higher landmark OS rates in the cardiovascular comorbidity subgroup rather than a genuine protective effect of having cardiovascular disease.

Although our post hoc analysis was exploratory and statistical subgroup comparisons were descriptive and for hypothesis-generating purposes only, these findings suggest that participants with cardiovascular or metabolic comorbidities generally experienced similar benefits in OS with STRIDE as those without comorbidities (in both the adjusted and unadjusted analyses), and there was no evidence that the presence of cardiovascular or metabolic comorbidities was predictive of treatment effect for OS.

Furthermore, participants treated with STRIDE experienced long-term survival, irrespective of the presence of comorbidities. For people with unresectable HCC, certain medical conditions and comorbidities may render patients less suitable for specific first-line therapies, particularly anti-angiogenic therapies, which may increase the risk for cardiovascular toxicity, hypertension, thrombosis, and bleeding [35, 36]. The STRIDE regimen offers a combination immunotherapy treatment option without conferring the toxicity profile of anti-angiogenic therapies.

The findings from a previous meta-analysis of other phase 3 trials evaluating immunotherapy in HCC suggested that people with metabolic dysfunction-associated steatohepatitis/MASLD HCC may derive less benefit from immunotherapy compared with those with HCC due to viral etiologies [37]. However, consistent with the present analysis, a recent post hoc analysis of the phase 3 IMbrave150 study showed that OS and PFS with atezolizumab plus bevacizumab were not consistent across liver disease etiologies [38]. The outcomes from HIMALAYA suggest a similar benefit in the setting of HCC with a nonviral etiology as in the overall HCC population, as demonstrated in a previous exploratory subgroup analysis of the HIMALAYA study, in which STRIDE and durvalumab monotherapy demonstrated an improved survival benefit vs. sorafenib in participants with underlying nonviral HCC [18, 39]. Since cardiovascular and metabolic comorbidities are common among people with nonviral HCC, our finding that participants with comorbidities experienced similar OS benefits with STRIDE to those without comorbidities was expected and supports previous nonviral etiology analyses in the HIMALAYA study [18, 38].

Moreover, the results of this exploratory, post hoc analysis suggest that STRIDE may also benefit participants with the type of comorbidities that predispose to or have been associated with MASLD [39]. Therefore, the presence of cardiovascular or metabolic comorbidities may not preclude the use of STRIDE in people with unresectable HCC who typically suffer from the respective conditions. As the number of people affected by MASLD is projected to reach 100.9 million cases by 2030, contributing to an increasing prevalence of comorbidities among people with HCC, the results reported in this analysis support the STRIDE regimen as a suitable treatment option for this patient population, irrespective of the presence of comorbidities [40, 41].

With respect to safety, the outcomes for STRIDE and durvalumab monotherapy compared with sorafenib in participants with cardiovascular or metabolic comorbidities were generally consistent with participants in the overall safety analysis set, with no new safety signals observed at the 4-year analysis [18]. In addition, regardless of treatment arms, participants with metabolic comorbidities were more likely to experience grade 3 or 4 TRAEs compared with all participants in the safety analysis set, although rates of grade 3 or 4 TRAEs were lower with STRIDE vs. sorafenib, consistent with the trend observed in all participants in the safety analysis set [18]. These findings highlight the challenges associated with the management of metabolic comorbidities during the treatment journey of participants with unresectable HCC.

The limitations of the HIMALAYA study have been discussed previously and include the open-label nature of the study design and the lack of stratification by geographic region [18]. In addition, this exploratory, post hoc analysis had no control for the type I error rate, and analysis of smaller not adequately powered subgroups resulted in wide CIs. Thus, results should be interpreted with caution, and the observed lack of significance between OS in comorbidity subgroups may not exclude the possibility of an effect. In addition, caution should be exercised when interpreting the tail of the Kaplan-Meier curves due to the small number of patients still at risk. Moreover, details of the comorbid conditions, such as severity or treatment, were not captured and therefore not considered in this analysis. Thus, the results of this exploratory, post hoc analysis may not be fully representative of all people with unresectable HCC with confounding comorbidities. Other limitations include that the cardiovascular and metabolic comorbidity subgroups mainly consisted of participants with hypertension and type 2 diabetes, respectively. Additionally, the HIMALAYA trial excluded participants who might be considered “high-risk” in clinical practice, including those with uncontrolled hypertension, main portal vein thrombosis, or active/prior gastrointestinal bleeding. Therefore, the results of our analyses may not be generalizable to people with unresectable HCC who have similar comorbidities in clinical practice. Lastly, the original randomized trial was not designed with the intention of assessing whether the comorbidities modify treatment effects; therefore, such analyses are purely exploratory, may not be able to be fully explained, and are susceptible to residual confounding.

These findings highlight the importance of considering cardiovascular and metabolic comorbidities when deciding on treatment for people with unresectable HCC. Taken together, these exploratory, post hoc analyses support a survival benefit and tolerable safety profile with STRIDE for people with unresectable HCC with cardiovascular or metabolic comorbidities, acknowledging the limitations reported above.

## Acknowledgments

The authors would like to thank the people who volunteered to participate in the HIMALAYA study, their families and loved ones, all the investigators and study site personnel, and the members of the Independent Data Monitoring Committee. The authors would also like to thank Alessandro Mascaro for their contributions to the statistical analyses reported in this manuscript. Medical writing support, under the direction of the authors, was provided by Robyn Johnson, MBBCh, Publications and Medical Affairs Division of Omnicom Health Medical Communications and was funded by AstraZeneca in accordance with Good Publication Practice (GPP 2022) guidelines.

## Statement of Ethics

The HIMALAYA study was conducted in accordance with the Declaration of Helsinki, International Council for Harmonisation Guideline for Good Clinical Practice, applicable regulatory requirements, and the AstraZeneca Bioethics Policy. Written, informed consent was obtained from participants or their legal representatives before participation. This study protocol was reviewed and approved by Ethics Committees at each of the participating sites. This full list of participating sites and Ethics Committees can be found in the Supplementary Materials of the 5-year updated analysis of the HIMALAYA study (Rimassa L, et al. *J Hepatol*. 2025;83(4):899–908).

## Conflict of Interest Statement

Lorenza Rimassa reports grant/research funding (to institution) from AbbVie, AstraZeneca, BeiGene, Bristol Myers Squibb, Exelixis, Fibrogen, Incyte, Ipsen, Jazz Pharmaceuticals, MSD, Nerviano Medical Sciences, Roche, Servier, Taiho Oncology, TransThera Sciences, and Zymeworks; consulting fees from AbbVie, AstraZeneca, Basilea, Bayer, Boehringer Ingelheim, Boston Scientific, Bristol Myers Squibb, Eisai, Elevar Therapeutics, Exelixis, Genenta, Guebert, Hengrui, Incyte, Ipsen, Jazz Pharmaceuticals, MSD, Nerviano Medical Sciences, Roche, Servier, Taiho Oncology, and Zymeworks; honoraria (lecture fees) from AstraZeneca, Bayer, Biologix, Bristol Myers Squibb, Eisai, Guerbet, Incyte, Ipsen, Roche, and Servier; and travel expenses from AstraZeneca and Servier. Lorenza Rimassa was also a member of the journal’s Editorial Board at the time of submission. Farshid Dayyani received consultant and research support (to the institution) from AstraZeneca; consulting fees from Eisai, Sirtex, and Taiho; and speaker fees from Astellas, BeOne, Ipsen, Sirtex, and Takeda. Robin K. Kelley reports consulting or advisory fees (to institution) from Agios, AstraZeneca, and MSD, and (to self) from Elevar, GSK, Jazz Pharmaceuticals, J Pharma, Moderna, Regeneron, and Tyra Biosciences; and research funding (to institution) from Agios, AstraZeneca, Bayer, Bristol Myers Squibb, Compass Therapeutics, Eli Lilly, EMD Serono, Exelixis, Genentech, Loxo Oncology, MSD, Novartis, Partner Therapeutics, QED, Relay Therapeutics, Roche, Servier, Surface Oncology, Taiho Pharmaceutical, and Tyra Biosciences. Bruno Sangro reports consulting or advisory fees from AstraZeneca, Bayer, Boston Scientific, Bristol Myers Squibb, Eisai, Incyte, Roche, Sirtex Medical, and Terumo; speaker fees from AstraZeneca, Eisai, Incyte, Roche, and Sirtex Medical; research funding (to institution) from Bristol Myers Squibb and Sirtex Medical; and being a steering committee member for AstraZeneca, Boston Scientific, Bristol Myers Squibb, and Roche. Stephen L. Chan reports advisory board fees from AstraZeneca, Eisai, and MSD; speaker fees from AstraZeneca, Bayer, Bristol Myers Squibb, Eisai, Elevar, Ipsen, MSD, and Roche; and research funding (to institution) from Bayer, Eisai, Ipsen, MSD, and Sirtex Medical. Stephen L. Chan was also a member of the journal’s Editorial Board at the time of submission. Hitomi Nakamura is an employee and shareholder of AstraZeneca. Lucy Clark is a Phastar employee contracted to AstraZeneca. Ioannis Xynos is an employee and shareholder of AstraZeneca. Ghassan K. Abou-Alfa reports grant/research funding from Arcus, AstraZeneca, BioNtech, Bristol Myers Squibb, Celgene, Flatiron, Genentech/Roche, Genoscience, Incyte, Polaris, Puma, QED, Silenseed, and Yiviva; and consulting fees from Adicet, Alnylam, AstraZeneca, Autem, BeiGene, Berry Genomics, Boehringer Ingelheim, Celgene, Cend, CytomX, Eisai, Eli Lilly, Exelixis, Flatiron, Genentech/Roche, Genoscience, Helio, Helsinn, Incyte, Ipsen, Merck, Nerviano, Newbridge, Novartis, QED, Redhill, Rafael, Servier, Silenseed, Sobi, Vector, and Yiviva. Ghassan K. Abou-Alfa also reports filed patent PCT/US2014/031545, filed: March 24, 2014, and priority application Serial No.: 61/804,907, filed: March 25, 2013.

## Funding Sources

This study was funded by AstraZeneca. AstraZeneca sponsored the trial, provided the trial drugs, and collaborated with the Steering Committee on the trial design and data collection, analysis, and interpretation. The manuscript was prepared and approved by all authors, with medical writing support funded by the sponsor. The sponsor reviewed and approved for submission the final draft of the manuscript to ensure accuracy of references to AstraZeneca products and clinical studies, guard against disclosure of confidential information, and check that information relevant to pending patents was handled appropriately.

## Author Contributions

Conception and design of the study: Lorenza Rimassa, Bruno Sangro, Stephen L. Chan, Hitomi Nakamura, Ioannis Xynos, and Ghassan K. Abou-Alfa. Data acquisition: Lorenza Rimassa, Farshid Dayyani, Robin K. Kelley, Bruno Sangro, Stephen L. Chan, and Ghassan K. Abou-Alfa. Analysis and interpretation of data: Lorenza Rimassa, Farshid Dayyani, Robin K. Kelley, Bruno Sangro, Stephen L. Chan, Hitomi Nakamura, Lucy Clark, Ioannis Xynos, and Ghassan K. Abou-Alfa. Administrative, technical, or material support: Ghassan K. Abou-Alfa. Study supervision: Robin K. Kelley, Bruno Sangro, and Ghassan K. Abou-Alfa. All authors contributed to the writing and reviewing of the manuscript, approved the final version, and agreed to be accountable for all aspects of the work in ensuring that questions related to the accuracy or integrity of any part of the article are appropriately investigated and resolved.

## Data Availability

Data underlying the findings described in this manuscript may be obtained in accordance with AstraZeneca’s data sharing policy described at https://astrazenecagrouptrials.pharmacm.com/ST/Submission/Disclosure. Data for studies directly listed on Vivli can be requested through Vivli at www.vivli.org. Data for clinical studies not listed on Vivli could be requested through Vivli at https://search.vivli.org/enquiries/link. AstraZeneca Vivli member page is also available outlining further details: https://vivli.org/ourmember/astrazeneca/.
